# Socio-demographic and antenatal care-related factors associated with early post-partum family planning use in Ethiopia: evidence from Ethiopian Demographic and Health Survey 2016 data

**DOI:** 10.3389/fgwh.2023.1131143

**Published:** 2023-09-01

**Authors:** Melaku Hunie Asratie, Getayeneh Antehunegn Tesema, Dagmawi Chilot, Fantu Mamo Aragaw, Mehari Woldemariam Merid, Daniel Gashaneh Belay

**Affiliations:** ^1^ Department of Women’s and Family Health, School of Midwifery, College of Medicine and Health Sciences, University of Gondar, Gondar, Ethiopia; ^2^ Department of Epidemiology and Biostatistics, Institute of Public Health, College of Medicine and Health Sciences, University of Gondar, Gondar, Ethiopia; ^3^ Center for Innovative Drug Development and Therapeutic Trials for Africa (CDTAfrica), Addis Ababa University, College of Health Sciences, Addis Ababa, Ethiopia; ^4^ Department of Human Physiology, University of Gondar, College of Medicine and Health Science, School of Medicine, Gondar, Ethiopia; ^5^ Department of Human Anatomy, College of Medicine and Health Sciences, University of Gondar, Gondar, Ethiopia

**Keywords:** early initiation, EDHS, Ethiopia, family planning, post-partum period

## Abstract

**Introduction:**

Initiation of family planning in the early post-partum period is a strategic move to reduce maternal, neonatal, and child mortality due to the negative consequences of short interbirth interval and the complications of unintended pregnancy. Antenatal care (ANC) is the noteworthy predictor of scaling up early initiation of post-partum family planning (PPFP) and preventing unintended pregnancy before menses resume. Despite the great role of ANC, information is scant about the effect of content, timing, and the number of ANC visits on the early initiation of PPFP in Ethiopia.

**Objective:**

This study aimed to assess the association of ANC services with the early initiation of PPFP in Ethiopia.

**Methods:**

The study was based on Ethiopian Demographic and Health Survey 2016 data, which was a cross-sectional survey from 18 January 2016 to 27 June 2016. A total weighted sample size of 2,920 post-partum women was included. A multilevel logistic regression model was used because of the hierarchical data, and variables with a *p*-value of ≤0.2 in the bivariable multilevel analysis were taken to multivariable multilevel analysis. An adjusted odds ratio with a 95% confidence interval (CI) was used to declare both the direction and strength of the association, and variables with a *p*-value of <0.05 were considered as statistically significant for the outcome variable.

**Results:**

The early initiation of PPFP was 20.4%. Women with at least four ANC visits [adjusted odds ratio (AOR) = 1.31; CI 1.12–2.32], women who started ANC within the first trimester (AOR = 1.25; CI 1.10–2.23), complete routine ANC (AOR = 1.11; CI 1.01–2.03), post-natal care (AOR = 1.45; CI 1.19–1.87), resumption of menses (AOR = 1.67; CI 1.18–1.93), urban residency (AOR = 2.14; CI 1.18–2.51), and high community women’s education (AOR = 1.71; CI 1.51–2.11) were variables significantly associated with the early initiation of PPFP.

**Conclusion:**

The early initiation of PPFP in Ethiopia was very low. Attention needs to be given to the quality of ANC, post-natal care, resumption of menses, residency, and community-level education of women to increase the prevalence of the early initiation of PPFP in Ethiopia. Therefore, the government should design a program targeting the quality of ANC in rural communities, considering women without menses and scaling up the education of women at the community level to the culture of the early initiation of PPFP in order to achieve reduced maternal, neonatal, and child mortality.

## Introduction

Provision of post-partum family planning (PPFP) services is one of the essential components of the maternal continuum of care that can be provided during labor and delivery and at any time of the post-partum period (1, 2). Currently, it is part of comprehensive maternal, neonatal, and child health services (3). In developing countries, PPFP provision is the selected strategy to halt maternal, neonatal, and child mortality (4). It is a means of making the post-partum period a high-impact and cost-effective program for the target group for maternal, neonatal, and child health improvements (5).

In Ethiopia, the majority of maternal and adolescent mortality is due to complications of unplanned and unwanted pregnancy (6, 7). Post-partum women have a high demand for spacing and limiting pregnancies. Approximately 75% of women have an unmet need for preventing pregnancy (6). The unmet need for post-partum contraceptive use is directly associated with unintended pregnancy, especially with the characteristics of short interbirth interval (8, 9). Evidence showed that more than 20% of births in developing countries are with short interbirth intervals (9, 10). A recently published article states that the prevalence of short interbirth intervals in Ethiopia is 46% (11). Developing countries, including Ethiopia, need to give attention to reducing this high magnitude of the short interbirth interval because it is one of the major causes of maternal, neonatal, infant, and child mortality (9–14). Specifically, short interbirth interval due to unmet need is associated with uteroplacental bleeding disorders (placental abruption, placenta previa, and vasa previa) which are the direct causes of maternal mortality (15). Children born within 24 months, especially the 12th and early months of an elder sibling, have a 60% higher risk of dying before celebrating their first birthday as compared to those born after an interval of 3–5 years. Another evidence suggested that children born with intervals of less than 2 years are at a 40% higher risk of dying (16). A study also showed that pregnancies conceived less than 20 months following a prior birth are at higher risk of early neonatal death, prematurity, fetal death, and low birth weight (17). In general, too closely spaced births due to an unmet need for PPFP pose substantial health risks for the mother, fetus, neonate, and child as well (18).

Attention should be given to the timing of initiation of post-partum contraceptives to avert the aforementioned challenges related to short interbirth intervals and the negative consequences of unintended pregnancy (19–22). Different kinds of literature recommend that post-partum contraceptives should be initiated in the early post-partum period (within 6 weeks) as much as possible (23–25). Early initiation of PPFP can avert 75% of maternal mortality, unintended pregnancy by two-thirds, and the risk of abortion by 73% (4). Additional evidence showed that early initiation of a post-partum contraceptive is the supreme intervention for the affirmation of optimal interbirth interval (26). The majority of the post-partum women become fecund before the resumption of the menstrual cycle (27); however, most women do not realize that they are at risk for subsequent pregnancy in this critical period (5). This is one of the hidden challenges in Ethiopia that needs attention to solve by cultivating a culture of the early initiation of PPFP among post-partum period women.

From the clinical experience of different scholars, they recommend that devoting time to counseling post-partum contraceptive use during the antenatal care (ANC) period is the preferred modality to the early initiation of PPFP. However, scant information about the effect of the content, timing, and the number of ANC visits on the early initiation of PPFP (within 6 weeks of post-partum women) in Ethiopia is found. With this paucity of high-quality evidence on the role of the quality of ANC, to improve the prevalence of the early initiation of PPFP, the real risk of short interbirth interval and unintended pregnancy with their negative consequences need great attention for fear of theoretical maternal, neonatal, and child risks. Therefore, this study aimed to assess the effect of content, timing, and the number of ANC visits on the early initiation of PPFP in Ethiopia. Furthermore, the findings of this study could guide program managers or policymakers for the improved prevalence of the early initiation of PPFP with the ultimate goal of reducing maternal, neonatal, and child death.

## Methods

### Study design, area, and period

A cross-sectional study survey was conducted among reproductive-aged women in Ethiopia from 18 January 2016 to 27 June 2016 by the Ethiopian Central Statistical Agency (ECSA). In the case of our study, we conducted a secondary analysis of the survey using the Ethiopian Demographic and Health Survey (EDHS) 2016. The EDHS 2016 was the fourth survey conducted among nine regional states of the country [Tigray; Afar; Amhara; Oromia; Somali; Benishangul-Gumuz; the Southern Nations, Nationalities, and Peoples’ Region (SNNPR); Gambella; and Harari regions] and two city administrations (Addis Ababa and Dire Dawa). Ethiopia is an eastern African country that is the second-most populous country after Nigeria. At the federal level, divided into nine regions and two city administrations for administrative purposes, those regions are subdivided into zones, zones divided into woredas, and woredas divided into kebeles (the lowest administrative unit). Kebeles is also divided into census enumeration areas (EAs).

The detail of the study area and study design was elaborated from the document of the Central Statistical Agency (CSA) of Ethiopia (28).

### Source population

All post-partum reproductive-aged women (15–48) who are current contraceptive users in Ethiopia were our source population.

### Study population

All post-partum period reproductive-aged women (15–48) currently using any contraceptive methods during the data collection period (18 January 2016 to 27 June 2016) in Ethiopia were the study population.

### Sample size determination and sampling procedure

In the EDHS, the survey was conducted with two stages; a stratified cluster sampling technique was used to select participants. In the first stage, 645 EAs were selected by stratifying into 202 urban areas and 443 rural areas. The sampling frame was the 2007 population and housing census using probability proportional to the EA scale. The details of the sampling procedure were elaborated in the EDHS 2016 report from the MEASURE DHS Program website (www.dhsprogram.com). Finally, the weighted values were used for the analysis in order to keep the representativeness of the sampled data. Women's record (IR) EDHS data sets were used. From this data set, we have fixed the study population by considering only PPFP users and non-pregnant women by using variables, and then, after the total sample size of the weighted data set that we have used in our study was 2,920 women who were post-partum, not pregnant, and currently using any kind of contraceptives. The variable month of starting a contraceptive was used to categorize the outcome variable, whether early initiation or late initiation.

### Study variables

**Dependent variable:** Early initiation of PPFP (yes/no).

**Independent variables:** All these independent variables were grouped into three major classifications (socio-demographic factors, obstetrical-related factors, and maternal healthcare service-related factors). During the analysis, age of women in a year, age of husband/partner, women's and husband’s level of education, religion, current working status, wealth status, relation to household head, exposure to mass media, parity, number of children under the age of 5 years, history of pregnancy termination, resumption menses, place of delivery, post-partum care, husband/partner desire for children, information about family planning services, number of visits by a health worker in the past 12 months, number of health facility visits in the last 12 months, and distance to health facility were variables moved as individual-level factors, whereas residency, community-level women's education, community-level poverty, and community-level media exposure were variables moved as community-level factors (Table 1).

**Table 1:** Description and measurement of independent variables.

Age of women	Re-coded into three categories with a value of “1” for 15–19 years, “2” for 20–34 years, and “3” for 35–49 years. In the data set, this variable was continuous data
Women’s level of education	The variable women's educational level was recorded as no education primary, secondary, and higher in the data set, and we used without change
Religion	Re-coded into four categories with a value of “1” for orthodox, “2” for Muslim, “3” for Protestant, and “4” for other religious groups (combining catholic, traditional, and the other religious categories as most women in this category are small in number)
Parity	In the data set, this variable was continuous data. We re-coded it into four categories with a value of “0” for nulliparous, “1” for primiparous, “2” for multiparous, and “3” for grand-multiparous
Information about family planning	This variable was generated from four variables from the data set: (1) heard about family planning from radio, (2) heard about family planning from newspaper/magazine, (3) heard about family planning from TV, and (4) heard about family planning from a text message. A women at least one from the four listed is considered as informed
Current working status	The variable current working status was recorded as yes and no in the data set and was used without change for this study
Wealth status	It was coded as “poorest,” “poorer,” “middle,” “richer,” and “richest” in the EDHS data set. For this study, we re-coded it into three categories as “poor” (includes the poorest and the poorer categories), “middle,” and “rich” (includes the richer and the richest categories)
Residence	The variable place of residence was recorded as “rural” and “urban” in the data set and was used without change for this study
Community media exposure	Defined as the proportion of women who had mass media exposure within the cluster. The aggregate of individual women with mass media exposure can show overall mass media exposure of the cluster. It was categorized as high if cluster has more than or equal to median proportion (57.14%) of women with mass media exposure or low otherwise
Community poverty	Defined as the proportion of women who resided in poor or poorest households within the cluster. The aggregate of individual households with poorest or poor wealth index can show overall poverty of the cluster. It was categorized as high if clusters had more than or equal to median proportion (60%) of poorest or poor households or low otherwise
Community women's education	Defined as the proportion of women who attended primary/secondary/higher education within the cluster. The aggregate of individual woman's primary/secondary/higher educational level can show overall educational attainment of the women in the cluster. It was categorized as high if clusters with more than or equal to median proportion (27.27%) of primary/secondary/higher education or low otherwise

### Operational definitions

**Early initiation of PPFP:** Prevention of unintended and closely spaced pregnancies within 6 weeks following childbirth using modern contraceptives (29).

**Components of routine ANC services:** This was an independent variable for this study that was measured based on eight essential elements of ANC services: blood pressure measurement, blood sample collection, urine sample collection, weight measurement, tetanus toxoid (TT2+) vaccination, iron folate (90+) supplementation, health education on danger signs and nutrition, and HIV testing. Information on these eight items of ANC content was derived from the response to the question “As part of your antenatal care during this pregnancy, were any of the following done at least once? Was your weight measured? Was your blood pressure measured?”. The answers were recorded as yes or no. It is possible that a single mother may have a urine test or blood test or measurement of weight or blood pressure several times during the same pregnancy. However, as the mother was asked to report any action at least once, the response for any action was recorded as a single action. On the basis of responses, we have created a composite index of ANC content as our second outcome variable, which comprises a simple count of the number of elements of care received. Finally, the outcome variable is dichotomized into incomplete if a woman gets less than eight services = 0 and complete if a woman gets all eight elements = 1 (30, 31).

**Post-partum period:** It is defined as the time limit from the delivery of the placenta to 12 months of the post-partum period. Therefore, all women within this time period were classified as “post-partum” (32).

### Data processing and statistical analysis

The data were first accessed from the website http://www.measuredhs.com/ by online request of permission through a detailed explanation of our research purpose, and the data were extracted and coded, and both descriptive and analytical analyses were done using statistical software STATA version 14. Statistical summaries such as proportion and median were used to present descriptive statistics.

EDHS data were collected by considering clusters as a study unit, and this violates the independent assumptions of a standard logistic regression model. Therefore, multilevel logistic regression analysis was implemented. The first intra-class correlation coefficient (ICC) of the null model was used to detect the presence of variation in the distribution of the outcome variable (early initiation of PPFP) among different clusters, and the magnitude was 22.5%, which entails that there is a significant clustering effect that should be considered during analysis using an advanced statistical model. The median odds ratio (MOR) was also another indicator of the presence of a significant clustering effect with the value of 2.52 (2.35–2.61) in the null model.

Fixed effects (a measure of association) were used to assess the relationship between the outcome variable and the independent variables. Crude odds ratio (COR) with a 95% confidence interval (CI) was used to measure both the direction and strength of the association. Variables with a *p*-value of ≤0.2 were selected for the analysis in the adjusted model. Finally, in the multilevel analysis, the association between the outcome variables and explanatory variables was judged by using an adjusted odds ratio (AOR) with respect to a CI, and statistical significance was declared at a *p*-value of <0.05.

Random effects (a measure of variability) were measured by ICC, MOR, proportion change in variance (PCV), and deviance (−2 log-likelihood ratio).

Intra-class correlation coefficient: ICC was the value used to detect the variation in the distribution of outcome variable (early initiation of PPFP) between clusters. In the null model, the ICC was 22.5%, which means, irrespective of other factors such as socio-demographic, obstetrical, and maternal healthcare service-related factors of our study, the cluster determined 22.5% of the variation in the distribution of the outcome variable.

Median odds ratio: MOR was used to quantify the middle odds ratio between the highest and the lowest odds ratios of the clustering effect. It is another way of quantifying cluster-level variance into odds ratio. The MOR in the null model of this study was 2.52 (2.35–2.61) which was significant. It was calculated as follows MOR = exp. [√ (2xVA) × 0.6745], MOR = e0.95√VA where VA = cluster-level variance.

Proportion change in variance: PCV was used to explain the percent of the variation in the early initiation of PPFP detected by the model with the available variables. The PCV of the final model of this study was 6%, which means that 6% of the variability was explained by the model that we fit, whereas the rest 94% of the variability was not explained by the model.

Deviance (−2 log-likelihood): Deviance was used to measure the total variations that come up with both the individual- and community-level factors. It was used to model comparison, and the model with the lowest deviance was taken for the interpretation of the finding, which was model IV.

### Ethical approval and consent to participate

Since the study was a secondary data analysis of publicly available survey data from the MEASURE DHS Program, ethical approval and participant consent were not necessary for this particular study. We requested the DHS Program, and permission was granted to download and use the data for this study from http://www.dhsprogram.com. The Institutional Review Board approved that procedures for DHS public-use data sets did not in any way allow respondents, households, or sample communities to be identified. No names of individuals or household addresses in the data file were recorded. The geographic identifiers only went down to the regional level (where regions are typically very large geographical areas encompassing several states/provinces). Each EA (primary sampling unit) had a PSU number in the data file, but the PSU numbers did not have any labels to indicate their names or locations.

## Results

### Socio-demographic characteristics of the study participants in Ethiopia

Among 2,920 post-partum women who were using contraceptives, 1,927 (66%) were within the age group of 20–34 years; 1,898 (65%) had a household head aged 31–59 years; 1,548 (53%) had no formal education; 1,139 (39%) had their husbands achieve primary education; and 2,599 (89%) of their household head’s sex were male. Among all participants, 1,489 (51%) were orthodox; 1,840 (63%) did not have work; 1,489 (51%) were rich; 2,219 (76%) were residing in rural areas; and 2,482 (85%) of them were the wives of the household head. Among all women who participated in this study, 1,956 (67%) and 1,840 (64%) were women with low community education and community media exposure, respectively, whereas 1,781 (61%) of the participants were at high community poverty (Table 2).

**Table 2:** Socio-demographic characteristics of study participants and their partners in Ethiopia.

Characteristics	Weighted frequency (*n* = 2,920)	Percent
Age of women in year		
15–19	146	5
20–34	1,927	66
35–49	847	29
Age of household head		
<31	818	28
31–59	1,898	65
>59	204	7
Women's level of education		
Had no formal education	1,548	53
Primary (grade 1–8)	905	31
Secondary (grade 9–12)	263	9
Higher	204	7
Husband’s/partner’s level of education		
Had no formal education	1,139	39
Primary (grade 1–8)	1,139	39
Secondary (grade 9–12)	350	12
Higher	292	10
Sex of household head		
Male	2,599	89
Female	321	11
Religion		
Orthodox	1,489	51
Muslim	613	21
Protestant	760	26
Others^a^	58	2
Current working status		
No	1,840	63
Yes	1,080	37
Wealth status		
Poor	818	28
Middle	613	21
Rich	1,489	51
Residency		
Urban	701	24
Rural	2,219	76
Relation to household head		
Head	322	11
Wife	2,482	85
Others^b^	116	4
Exposure to mass media		
Yes	1,577	54
No	1,343	46
Community women's education		
Low	1,956	67
High	964	33
Community poverty		
Low	1,139	39
High	1,781	61
Community media exposure		
Low	1,840	63
High	1,080	37

^a^ Others = Catholic, traditional followers.

^b^ Others = daughter, sister.

### Maternal healthcare service-related characteristics of participants in Ethiopia

Among all participants, 1,956 (67%) had fewer than four ANC visits; 1,635 (56%) started their ANC visits within the first trimester, and 1,752 (60%) did not receive the complete components of routine ANC services. Of all respondents, 2,132 (73%) reported that distance was a significant problem when reaching the nearby health facility; 1,986 (68%) did not have a chance to be visited for fieldwork; and 1,548 (53%) had information about family planning. Among all participants, 2,336 (80%) delivered at a health facility; 2,190 (75%) had post-partum care; and 1,635 (56%) did not visit a health facility within 12 months (Table 3).

**Table 3:** Maternal healthcare service-related characteristics of participants in Ethiopia (*n* = 2,920).

Variables	Frequency	Percent
Number of antenatal care visit		
Less than four visits	1,956	67
Four and above visits	964	33
Gestational age for the first antenatal care visit		
Within the first trimester	1,635	56
Within the second trimester and above	1,285	44
Content of routine antenatal care		
Did not complete routine antenatal care	1,752	60
Completely got routine antenatal care	1,168	40
Distance to reach the nearby health facility		
Was a big problem	2,132	73
Was not a big problem	788	27
Visited by field worker		
No	1,986	68
Yes	934	32
Information about family planning		
No	1,372	47
Yes	1,548	53
Place of delivery		
Home	584	20
Health facility	2,336	80
Post-partum care		
No	730	25
Yes	2,190	75
Visited health facility within 12 month		
No	1,635	56
Yes	1,285	44

### Obstetrical-related characteristics of participants in Ethiopia

Among the 2,920 participants, 1,285 (44%) were multiparous; 1,680 (58%) had started menstruation, and 1,723 (59%) had a number of children under 5 years old. Of all participants, 2,570 (88%) had no bad obstetric history [stillbirth and abortion (both induced and spontaneous)], and 1,110 (38%) of their husbands wanted more children (Table 4).

**Table 4:** Obstetrical-related characteristics of participants in Ethiopia (*n* = 2,920).

Variables	Frequency	Percent
Parity		
Primiparous	584	20
Multiparous	1,285	44
Grand-multiparous	1,051	36
Menses resumed		
No	1,240	42
Yes	1,680	58
Number of children aged under 5 years		
1	1,723	59
2	876	30
≥3	321	11
History of pregnancy termination		
No	2,570	88
Yes	350	12
Husband’s desire for children		
Both want same	1,022	35
Husband wants more	1,110	38
Husband wants fewer	204	7
Do not know	584	20

1 = the prevalence of early initiation.

2 = the prevalence of not early initiated.

### The prevalence of post-partum family planning

**Figure 1:**
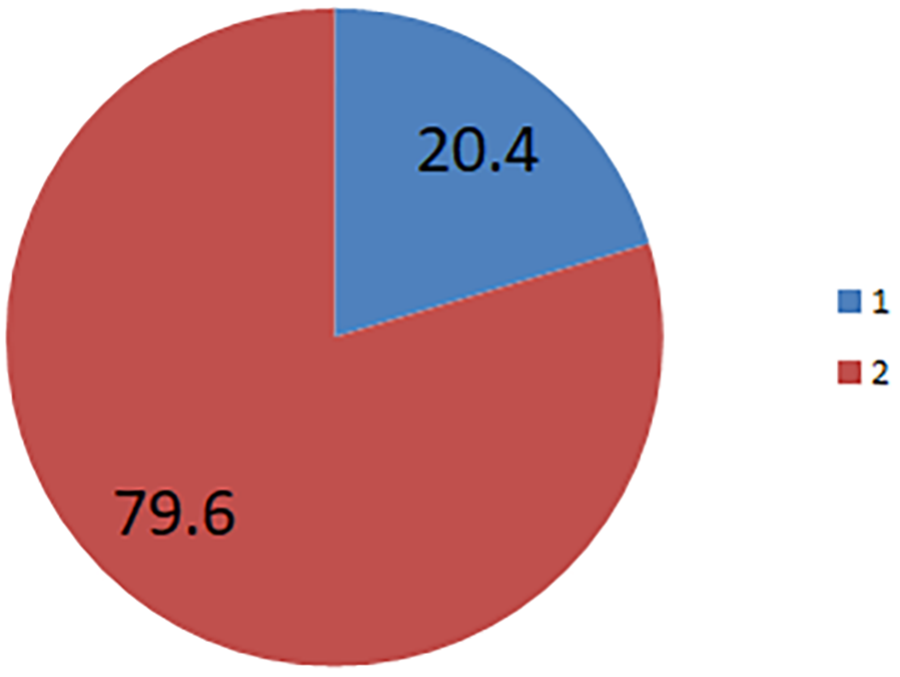
The prevalence of early initiation of post-partum family planning in Ethiopia.

The prevalence of the early initiation of PPFP was 20.4%; 95% (19.25–21.7%]) (Figure 1).

### Factors associated with early initiation of post-partum family planning

A total of three models were fitted to assess the association of variables. In model II, eight individual-level variables and, in model III, two community-level variables were significantly associated with the outcome variable. When we come to model IV, a total of seven variables (number of ANC, gestational age for the first ANC visit, components of routine ANC, post-partum care, resumption of menses, residency, and community women's education) were statistically significant with the early initiation of PPFP.

Women with at least four ANC visits were 1.31 times more likely to initiate PPFP (AOR = 1.31; 95% CI 1.12–2.32) as compared with those with ANC follow-up of less than four. Likewise, women who started ANC within the first trimester were 1.25 times more likely to initiate early PPFP (AOR = 1.25; 95% CI 1.10–2.23) compared with those who had started their ANC visit in the second trimester and above gestational age. Women who had received the full components of routine ANC were 1.11 times more likely to initiate early PPFP (AOR = 1.11; 95% CI 1.01–2.03) compared with those who did not get full components of routine ANC.

The odds of the early initiation of PPFP was 1.45 times higher among women with post-partum care (AOR = 1.45; 95% CI 1.19–1.87) compared with those who had no post-partum care. Women with menses resumption at the time of the survey were 1.67 times more likely to initiate early PPFP (AOR = 1.67; 95% CI 1.18–1.93) as compared with those who never had their menses resumed. The odds of early initiation of PPFP was 2.14 times higher among women who reside in urban areas (AOR = 2.14; 95% CI 1.18–2.51) as compared with those residing in rural areas. The odds of the early initiation of PPFP was 1.71 times higher among women with high community education (AOR = 1.17; 95% CI 1.51–2.11) as compared with those who had low community education (Table 5).

**Table 5:** Multivariable multilevel logistic regression analysis of factors associated with early initiation of postpartum family planning in Ethiopia (*n* = 2,920).

Variable	Null model	Model II	Model III	Model IV
		AOR (95% CI)	AOR (95% CI)	AOR (95% CI)
Number of antenatal care				
Less than four visits		1		1
Four and above visits		1.48 (1.22–2.18)	1.42 [1.11–2.11]	1.31 (1.12–2.32)***
Antenatal care start				
Within the first trimester		1.51 (1.23–2.25)	1.42 [1.24–2.54]	1.25 (1.10–2.23)***
Within the second trimester and above		1	1	1
Content of antenatal care				
Not complete routine ANC		1	1	1
Complete routine ANC		1.64 (1.12–2.42)	1.27 [1.11–2.75]	1.11 (1.01–2.03)***
Age of women				
15–19		1		1
20–34		2.01 (0.81–2.65)		1.22 (0.66–1.81)
35–49		1.5 (0.57–2.52)		1.14 (0.77–1.73)
Age of household head				
<31		1		1
31–59		0.29 (0.13–1.12)		0.88 (0.65–1.05)
>59		0.45 (0.26–1.54)		0.45 (0.22–5.69)
Women’s educational level				
No formal education		1		1
Primary (grades 1–8)		1.35 (0.52–1.75)		1.55 (0.22–1.91)
Secondary(grades 9–12)		2.11 (0.88–2.61)		1.97 (0.98–2.95)
Higher		1.72 (0.81–2.19)		1.19 (0.78–2.51)
Religion				
Orthodox		1		1
Muslim		2.23 (0.82–2.89)		1.11 (0.78–1.57)
Protestant		1.45 (0.66–1.79)		1.75 (0.87–2.22)
Others*		2.14 (0.6–2.79)		1.69 (0.88–2.79)
Wealth status				
Poor		1		1
Middle		1.25 (0.85–3.16)		1.16 (0.91–1.58)
Rich		2.49 (1.83–2.18)		1.49 (0.78–1.86)
Post-partum care				
No		1		1
Yes		2.23 (1.85–1.86)		1.45 (1.19–1.87)***
History of termination pregnancy				
No		1		1
Yes		2.18 (1.65–3.16)		0.83 (0.65–1.19)
Information about family planning				
No		1		1
Yes		0.78 (0.42–1.84)		0.92 (0.77–1.25)
Visited by field worker				
No		1		1
Yes		2.9 (1.56–3.32)		1.14 (0.91–1.39)
Menses resumption				
No		1		1
Yes		2.81 (2.8–3.24)		1.67 (1.18–1.93)***
Residency				
Rural			1	1
Urban			2.23 (1.74–3.21)	2.14 (1.18–2.51)**
Community women’s education				
Low			1	1
High			2.29 (1.61–2.78)	1.71 (1.51–2.11)***
Community poverty				
Low			1	
High			0.75 (0.36–1.77)	0.86 (0.78–1.19)
Community media exposure				
Low			1	1
High			0.78 (0.35–1.52)	1.17 (0.87–2.10)
Random effect				
Community-level variance	0.95	0.92	0.90	0.89
ICC	22.5%	21.9%	21.5%	21.3%
MOR	2.52 (2.35–2.61)	2.48	2.46	2.45
PCV	Reference	3%	5%	6%
Model fit statistics				
Log likelihood	−2,032.431	−1,961.9,667	−2,026.5,522	−1,944.3,158
Deviance	4,064.862	3,923.9,334	4,053.1,044	3,888.6,316

AOR, adjusted odds ratio; CI, confidence interval; 1, reference category; *0.05 < *p* < 0.2, **0.001 < *p* < 0.05, ***p < 0.001.

Model II: includes all individual-level factors and number of antenatal care, start of antenatal care, and content of antenatal care.

Model III: includes all community-level factors and number of antenatal care, start of antenatal care, and content of antenatal care.

## Discussion

Designing, testing, and scaling up effective, affordable, and sustainable health interventions in low-resource countries are critical to address the high unmet need for PPFP (28). The study finds that the magnitude of the early initiation of PPFP is 20.4%. This is lower than that reported in previous studies conducted in Southern Ethiopia (31.7%) (33), Dilla town (Southern Ethiopia) (39.2%) (34), Tanzania (37%) (35), and Uganda (52%) (24). The possible explanation for the low prevalence of the early initiation of PPFP in the current study compared with two studies conducted in Southern Ethiopia could be due to the variation in the composition of the study population. This means that the current study is based on EDHS data which includes a larger proportion of rural communities and women in the rural community less likely to initiate early PPFP. This hypothesis is also supported by the current study as residency is significantly associated with the early initiation of PPFP. The possible explanation for the low prevalence of the current study compared with the previous studies conducted in Tanzania and Uganda could be the difference in socio-demographic factors among the study populations and the difference in the time trend of the data collected.

Regarding the factors associated with the early initiation of PPFP, the number of ANC services, gestational age for the first ANC visit, and content of routine ANC services had a great effect on the early initiation of PPFP. Antenatal care is a noteworthy predictor of subsequent adherence to the maternity continuum of care (health facility delivery, post-natal care, and PPFP use) (36, 37). Evidence shows that the quality of antenatal care is a driving force to achieve the sustainable development goal related to the reduction of maternal, neonatal, and child mortality (38–40). On the other hand, maternal, neonatal, and child mortality are secondary to short interbirth intervals, and the negative consequence of unintended pregnancy is still a problem in Ethiopia which can be alleviated by respecting the quality of maternal healthcare services, especially antenatal care services. The coverage of at least one antenatal care visit in Ethiopia has reached 62% from the EDHS 2016 report (41). This improvement did not result in a significant upswing in maternal, neonatal, and child health. This unresponsive challenge with the existing improved antenatal care service coverage might be solved with an approach to qualified antenatal care services. However, evidence related to the impact of the quality of antenatal care has not been explained well yet. Therefore, this study needs to examine the effect of the quality of antenatal care (number of visits, timing of antenatal care start, and the contents of routine care provided during the antenatal care period) on the early initiation of PPFP.

Women with at least four or more ANC visits were 1.31 times more likely to early initiate PPFP as compared with those who had antenatal care visits of less than four. This finding was supported by different studies (34, 42–47). The possible explanation could be that women with a greater number of antenatal care visits have greater contact with healthcare providers. This can be important to get the full service of antenatal care including counseling about birth spacing and early initiation of PPFP service. In addition, it is obvious that family planning use-related counseling is recommended after the third trimester or more at the fourth visit (37, 47). Therefore, those women with four and more antenatal care visits might have better information about PPFP use, and they might initiate early within 6 weeks of the post-partum period compared with those with less than four antenatal care visits.

Women who started ANC within the first trimester were 1.25 times more likely to initiate early PPFP as compared with women's first antenatal care visit starting within the second trimester and above. This finding was supported by studies conducted in Cambodia and Tanzania (46, 48). The possible explanation for this could be that women who start antenatal care at an early gestational age have a higher chance to contact healthcare providers and they could get PPFP counseling services from this intern, who helps them initiate PPFP early. The other possible explanation which is supported by evidence is that women who start antenatal care at an early gestational age are mostly free from the cultural influence from the community to attend the maternity continuum of care (49). Reduced exposure to misleading rumors about post-partum contraceptives might encourage women to initiate use of contraceptives earlier as compared to women influenced solely by cultural beliefs.

Lastly, women who completely took routine antenatal care services were 1.11 times more likely to initiate early PPFP compared with those who did not get full components of routine antenatal care services. Components of routine antenatal care services are the crux part of antenatal care, which is provided for all pregnant women irrespective of their risk/complication status (50, 51). Therefore, those services directly measure both the commitment of the healthcare provider assigned for antenatal care provision and women's engagement across the maternity continuum of care (52). Women who are committed to attending the full components of routine antenatal care services might be more likely to engage in the subsequent elements of the maternity continuum of care including the early initiation of PPFP compared with those women who did not receive the full components of routine antenatal care (53).

Four additional variables (post-partum care, resumption of menses, residency, and community women's education) statistically significant with the outcome variable early initiation of PPFP were identified. Women with post-partum care were 1.45 times more likely to initiate PPFP as compared with those who had no post-partum care. This finding was supported by different studies conducted in Ethiopia and Kenya (33, 54, 55). The possible explanation could be that post-partum care is one of the elements of the maternity continuum of care and routinely recommends PPFP use (56). Therefore, those women with post-partum care have greater information about the timing of post-partum contraceptive use, and they might initiate PPFP early compared with women without post-partum care.

In this study, the odds of early initiation of PPFP was 1.67 higher in women whose menses had resumed. This finding was supported by the study conducted in western Ethiopia as women with menses resumption of 2.6 times a higher time (within 6 weeks of the post-partum period) initiate PPFP compared with those who have menses yet not resumed (33). Another study conducted in Ethiopia showed that resumption of menses is significantly associated with the early initiation of PPFP (34). The possible explanation could be due to menses, which is alarming because of the occurrence of pregnancy whenever there is sexual intercourse in the post-partum period (57). Evidence showed that the majority of post-partum period women depended on their menses resumption to initiate PPFP and they were not informed about the occurrence of pregnancy with the absence of menses (58, 59). This was one of the challenges in Ethiopia for the huge number of unintended pregnancies due to post-partum contraceptives not used (60–62).

The odds of early initiation of PPFP were 2.14 times higher among women residing in urban areas compared with their counterparts. This finding was supported by different studies as residence was significantly associated with PPFP use (63, 64). The possible explanation could be that those women who reside in urban areas are nearest to media exposure and most media has a positive impact on awareness creation about family planning use (65). On the other hand, urban areas are known for a large proportion of educated participants (66), and education is one factor for the early initiation of PPFP in Ethiopia as proven by the current study.

Women with a high level of community education were 1.71 times more likely to initiate PPFP early compared with their counterparts. This finding was supported by evidence conducted in southern Ethiopia as women with high-level educational status were 1.5 times more likely to initiate PPFP early (34). The possible explanation could be that education is the driving force to be engaged on the continuum of maternity care including PPFP use in Ethiopia (67, 68). Educated women are familiar with the methods of contraceptives that are suitable for the post-partum period, and they might initiate PPFP early. The other possible explanation would be that educated women might be informed that pregnancy can occur before post-partum menses resumption and they might use contraceptives in the early post-partum period due to fear of unintended pregnancy.

## Strengths and limitations of this study

This study used nationally representative data, which were collected with standardized and validated data collection tools.

This study used an advanced model that accounts for the correlated nature of the EDHS data in the determination of estimates.

The cross-sectional nature of the survey does not show the temporal or causal relationship between independent variables and the outcome variables.

The EDHS data were based on post-partum period women with the last birth at the different time periods, and it might cause recall bias on the content of antenatal care services and timing of the initiation of PPFP.

The initiation of PPFP within 6 weeks of post-partum period included women who had initiated even within 2 months. This might increase the prevalence of the outcome variable.

## Conclusions and recommendations

This study demonstrated that early initiation of PPFP was low. Women with at least four ANC visits, first antenatal care visit within the first trimester, the full content of routine antenatal care services, having post-partum care, resumed menses, urban residency, and high community education were positively associated with early initiation of PPFP. The Demographic and Health Survey should revise the way of reaching out to the timing of PPFP initiation in weeks rather than in months.

## Data Availability

The original contributions presented in the study are included in the article, and further inquiries can be directed to the corresponding author.
